# Supplementary material for: Beliefs are multidimensional and vary in stability over time - psychometric properties of the Beliefs and Values Inventory (BVI)
Source: PeerJ. 2019 Apr 25;7:e6819. doi: 10.7717/peerj.6819 (PMC6487186; doi:10.7717/peerj.6819)
Supplement: Appendix A — The Beliefs and Values Inventory (55 items) assess beliefs across themes of science, politics, the paranormal, religion, and morality, by dimensions of agreement, interest, and self-relevance. In addition, there are 5 control questions. [file peerj-07-6819-s001.docx]

**Beliefs and Values Inventory**

The Belief and Values Inventory aims to capture the agreement, perceived self-relevance, and interest that individuals place on the following statements. The questionnaire spans political, scientific, religious, paranormal, and moral statements, as well as including 5 control questions.

Participants are asked after each statement to rate their **Agreement, Self-Relevance, and Interest** on a scale of **1-10**.

| **Theme** | **General/ Specific** | **Question** |
| --- | --- | --- |

| **Politics** | General  (Government responsibility and style) | 1. The government or state has responsibility to look after the wellbeing of the population. |
| --- | --- | --- |
|  |  | 1. Democracy is the best way to govern a country. |
|  |  | 1. The government or state should have as little involvement in the lives of the population as possible. |
|  |  | 1. Taxation should be high to provide high quality public services. |
|  |  | 1. High tax rates penalize people and families who’ve worked hard to earn their money. |
|  | Specific  (Political ideology) | 1. It’s good that a lot of countries and states have legalized abortion. |
|  |  | 1. Free healthcare for all, irrespective of income, is a fundamental human right. |
|  |  | 1. It’s right to legally allow people of the same sex to get married. |
|  |  | 1. Women should be legally entitled to equal pay to men for the same work. |
|  |  | 1. Severe punishment for selling and using drugs is the best way to prevent new users. |

| **Morality** | General | 1. It’s ok to hit or physically hurt someone for no reason if no one is around to know it happened. |
| --- | --- | --- |
|  | (General moral transgressions) | 1. Making a personal attack on someone on social media is ok if your identity is concealed. |
|  |  | 1. Saying something to cause emotional distress is fine if there aren’t any negative personal consequences. |
|  |  | 1. It’s fine to never share your money if there were no negative personal consequences. |
|  |  | 1. It is always wrong to have sexual contact with someone if they’re unable to give consent. |
|  | Specific | 1. Running a red traffic light is fine if no one is around |
|  | (Specific convention) | 1. Spitting on the street is wrong, even if no one sees you do it. |
|  |  | 1. Drink-driving isn’t a problem as long as no one gets hurt. |
|  |  | 1. Skipping a queue is never ok, even if no one notices. |
|  |  | 1. Not paying for a bus/train is fine as long as no one catches you. |

| **Science** | General | 1. Science will never fully understand the mysteries of the universe. |
| --- | --- | --- |
|  | (Belief in the power of general scientific enquiry) | 1. Reason and measurement are the only reliable basis for knowledge. |
|  |  | 1. Science will eventually give a more correct and reliable account of human behavior than literary works such as novels, plays, and poetry. |
|  |  | 1. The scientific method is the best technique we have to understand the way we should live our lives. |
|  |  | 1. Science is our most powerful tool for improving human health. |
|  | Specific | 1. Cigarette smoking causes lung cancer. |
|  | (Belief in specific factual evidence) | 1. There is no evidence that vaccines cause autism. |
|  |  |  |
|  |  | 1. Carbon dioxide emissions due to human activity are an important cause of global warming. 2. Homeopathy is as effective as modern medical treatments. |
|  |  | 1. Evolutionary theory is the best explanation we have for the origin of species. |

| **Religion** | General | 1. Each human being has a soul or spirit which survives death. |
| --- | --- | --- |
|  | (Core religious metaphysical principles) | 1. God(s) can intervene in the world to influence natural processes |
|  |  | 1. Death is not the end of personal existence. |
|  |  | 1. God(s) only exists in the human imagination (r) |
|  |  | 1. Living a good life will mean rewards in the next. |
|  | Specific | 1. Prayer or religious practice is one of the most important ways of improving our lives. |
|  | (Belief in religious application) |  |
|  |  |  |
|  |  | 1. God can communicate with people, but people often mistake the message. 2. Practicing religion makes people more moral than atheists. 3. Religion should play a central role in society. |
|  |  | 1. If religions were properly practiced the world would be a better place. |

| **Paranormal** | Specific | 1. A person’s star sign gives important information about their character |
| --- | --- | --- |
|  | (Magical thinking) |  |
|  |  | 1. The number 13 is unlucky. |
|  |  | 1. Tarot cards can genuinely predict the future. 2. Misalignment of chakras can cause ill health. |
|  |  | 1. Crystals can be used for healing. |
|  |  |  |
|  | General  (Clinical analogues) | 1. It is possible to communicate with people after they’ve died. |
|  |  |  |
|  |  |  |
|  |  | 1. Certain objects and actions can ward off bad luck. |
|  |  | 1. Some people have special powers to affect things at a distance. |
|  |  | 1. You can learn about your destiny through hidden signs in the world around us. |
|  |  | 1. Some people can affect the world around them purely through mental activity. |

| **Control Questions** |  | 1. The earth revolves around the sun. |
| --- | --- | --- |
|  |  | 1. Barack Obama was previously president of the USA. |
|  |  | 1. Murdering someone is punishable under criminal law in the UK. |
|  |  | 1. History is a study of the past. |
|  |  | 1. Australia is located in the southern hemisphere. |
