# Supplementary material for: Beliefs are multidimensional and vary in stability over time - psychometric properties of the Beliefs and Values Inventory (BVI)
Source: PeerJ. 2019 Apr 25;7:e6819. doi: 10.7717/peerj.6819 (PMC6487186; doi:10.7717/peerj.6819)
Supplement: Appendix B — The first table depicts total Item loading by name and factor (loading threshold = 0.4; bold = 0.5 threshold; Red = Politics, Green = Science, Blue = Paranormal, Purple = Religion, Orange = Morality). The next three tables depict individual dimension factor loadings. [file peerj-07-6819-s002.docx]

| Factor | Items |
| --- | --- |
| 1 | **P_G1A, P_G2A, P_G3A, P_G4A, P_G5A, P_S1A, P_S2A, P_S3A, P_S4A, P_S5A, P_G1R, P_G2R, P_G3R, P_G4R, P_G5R, P_S1R, P_S2R, P_S3R, P_S4R, P_S5R, P_G1I, P_G2I, P_G3I, P_G4I, P_G5I, P_S1I, P_S2I, P_S3I, P_S4I, P_S5I,** R_G1A, R_G2A, R_G3A, S_S4A |
| 2 | **R_G1A, R_G2A, R_G3A, R_G4A, R_G5A, R_S1A, R_S2A, R_S3A, R_S4A, R_S5A, R_G1R, R_G2R, R_G3R, R_G5R, R_S1R, R_S2R, R_S3R, R_S4R, R_S5R, R_G1I, R_G2I, R_G3I, R_G5I, R_S1I, R_S2I, R_S3I, R_S4I, R_S5I, Pol_S3A,** Pol_S1I, **S_S5A** |
| 3 | **S_G2A, S_G4A, S_G5A, S_S5A, S_G2R, S_G4R, S_G5R, S_S5R,** S_G1I**, S_G2I, S_G3I, S_G4I,** S_S5I |
| 4 | **M_G1I, MG1R, M_G2I, M_G2R, M_G3I, M_G3R, M_S1I, MS1R, M_S3I,** M_S5R, M_S5I |
| 5 | **M_G5I, M_G5R, Pol_S1I, Pol_S1R,** Pol_S3I, Pol_S3R, **Pol_S4I, Pol_S4R**, S_S1I, S_S1R, S_S2R |
| 6 | Pol_G1A, **Pol_G1I, Pol_G1R, Pol_G2I, Pol_G3I, Pol_G3R, Pol_G4I, Pol_S2I,** Pol_S2R |
| 7 | **M_S2I, M_S2R,** M_S4I, M_S4R |
| 8 | M_G3A, M_S1A, M_S3A |
| 9 | Pol_G4A, Pol_G5A |
| 10 | Pol_G2A, Pol_G2R, Pol_G2I |

| Interest | MR1 | MR3 | MR2 | MR4 | MR5 | MR6 | MR7 |
| --- | --- | --- | --- | --- | --- | --- | --- |
| S_G1_I |  |  | 0.533 |  |  |  |  |
| S_G2_I |  |  | 0.604 |  |  |  |  |
| S_G3_I |  |  | 0.536 |  |  |  |  |
| S_G4_I |  |  | 0.706 |  |  |  |  |
| S_G5_I |  |  | 0.484 |  |  |  |  |
| S_S1_I |  |  |  |  | 0.451 |  |  |
| S_S2_I |  |  |  |  | 0.448 |  |  |
| S_S3_I |  |  |  |  |  |  |  |
| S_S4_I | 0.421 |  |  |  |  |  |  |
| S_S5_I |  |  | 0.612 |  |  |  |  |
| R_G1_I | 0.484 | 0.519 |  |  |  |  |  |
| R_G2_I |  | 0.788 |  |  |  |  |  |
| R_G3_I |  | 0.512 |  |  |  |  |  |
| R_G4_I |  |  |  |  |  |  |  |
| R_G5_I |  | 0.605 |  |  |  |  |  |
| R_S1_I |  | 0.849 |  |  |  |  |  |
| R_S2_I |  | 0.777 |  |  |  |  |  |
| R_S3_I |  | 0.590 |  |  |  |  |  |
| R_S4_I |  | 0.708 |  |  |  |  |  |
| R_S5_I |  | 0.656 |  |  |  |  |  |
| P_G1_I | 0.700 |  |  |  |  |  |  |
| P_G2_I | 0.729 |  |  |  |  |  |  |
| P_G3_I | 0.619 |  |  |  |  |  |  |
| P_G4_I | 0.733 |  |  |  |  |  |  |
| P_G5_I | 0.593 |  |  |  |  |  |  |
| P_S1_I | 0.701 |  |  |  |  |  |  |
| P_S2_I | 0.631 |  |  |  |  |  |  |
| P_S3_I | 0.772 |  |  |  |  |  |  |
| P_S4_I | 0.656 |  |  |  |  |  |  |
| P_S5_I | 0.724 |  |  |  |  |  |  |
| Pol_S1_I |  |  |  |  | 0.557 |  |  |
| Pol_S2_I |  |  |  |  |  |  |  |
| Pol_S3_I |  |  |  |  | 0.481 |  |  |
| Interest | *MR1* | *MR3* | *MR2* | *MR4* | *MR5* | *MR6* | *MR7* |
| Pol_S4_I |  |  |  |  | 0.615 |  |  |
| Pol_S5_I |  |  |  |  |  |  |  |
| Pol_G1_I |  |  |  |  |  |  | 0.415 |
| Pol_G2_I |  |  |  |  |  |  |  |
| Pol_G3_I |  |  |  |  |  |  |  |
| Pol_G4_I |  |  |  |  |  |  |  |
| Pol_G5_I |  |  |  |  |  |  |  |
| M_G1_I |  |  |  | 0.737 |  |  |  |
| M_G2_I |  |  |  | 0.632 |  |  |  |
| M_G3_I |  |  |  | 0.621 |  |  |  |
| M_G4_I |  |  |  |  |  |  |  |
| M_G5_r_I |  |  |  |  | 0.616 |  |  |
| M_S1_I |  |  |  | 0.536 |  |  |  |
| M_S2_r_I |  |  |  |  |  | 0.552 |  |
| M_S3_I |  |  |  | 0.641 |  |  |  |
| M_S4_r_I |  |  |  |  |  | 0.497 |  |
| M_S5_I |  |  |  | 0.428 |  |  |  |

| Relevance | MR1 | MR3 | MR4 | MR2 | MR5 | MR7 | MR6 |
| --- | --- | --- | --- | --- | --- | --- | --- |
| S_G1_R |  |  |  |  |  |  |  |
| S_G2_R |  |  |  | 0.671 |  |  |  |
| S_G3_R |  |  |  | 0.511 |  |  |  |
| S_G4_R |  |  |  | 0.676 |  |  |  |
| S_G5_R |  |  |  | 0.437 |  |  |  |
| S_S1_R |  |  |  |  |  | 0.416 |  |
| S_S2_R |  |  |  |  | 0.426 |  |  |
| S_S3_R |  |  |  |  |  |  |  |
| S_S4_R | 0.418 |  |  |  |  |  |  |
| S_S5_R |  |  |  | 0.572 |  |  |  |
| R_G1_R | 0.441 | 0.580 |  |  |  |  |  |
| R_G2_R |  | 0.820 |  |  |  |  |  |
| R_G3_R |  | 0.563 |  |  |  |  |  |
| R_G4_R |  |  |  |  |  |  |  |
| R_G5_R |  | 0.630 |  |  |  |  |  |
| R_S1_R |  | 0.847 |  |  |  |  |  |
| R_S2_R |  | 0.790 |  |  |  |  |  |
| R_S3_R |  | 0.514 |  |  |  |  |  |
| R_S4_R |  | 0.697 |  |  |  |  |  |
| R_S5_R |  | 0.664 |  |  |  |  |  |
| P_G1_R | 0.631 |  |  |  |  |  |  |
| P_G2_R | 0.719 |  |  |  |  |  |  |
| P_G3_R | 0.591 |  |  |  |  |  |  |
| P_G4_R | 0.700 |  |  |  |  |  |  |
| P_G5_R | 0.565 |  |  |  |  |  |  |
| P_S1_R | 0.721 |  |  |  |  |  |  |
| P_S2_R | 0.571 |  |  |  |  |  |  |
| P_S3_R | 0.767 |  |  |  |  |  |  |
| P_S4_R | 0.673 |  |  |  |  |  |  |
| P_S5_R | 0.753 |  |  |  |  |  |  |
| Pol_S1_R |  |  |  |  | 0.616 |  |  |
| Pol_S2_R |  |  |  |  |  |  | 0.480 |
| Pol_S3_R |  |  |  |  | 0.421 |  |  |
| Pol_S4_R |  |  |  |  | 0.462 |  |  |
| Pol_S5_R |  |  |  |  |  | 0.561 |  |
| Relevance | *MR1* | *MR3* | *MR4* | *MR2* | *MR5* | *MR7* | *MR6* |
| Pol_G1_R |  |  |  |  |  |  | 0.472 |
| Pol_G2_R |  |  |  |  |  |  | 0.400 |
| Pol_G3_R |  |  |  |  |  |  |  |
| Pol_G4_R |  |  |  |  |  |  |  |
| Pol_G5_R |  |  |  |  |  |  |  |
| M_G1_R |  |  | 0.694 |  |  |  |  |
| M_G2_R |  |  | 0.591 |  |  |  |  |
| M_G3_R |  |  | 0.591 |  |  |  |  |
| M_G4_R |  |  |  |  |  |  |  |
| M_G5_r_R |  |  |  |  | 0.477 |  |  |
| M_S1_R |  |  | 0.571 |  |  |  |  |
| M_S2_r_R |  |  |  |  |  | 0.497 |  |
| M_S3_R |  |  | 0.647 |  |  |  |  |
| M_S4_r_R |  |  |  |  |  |  |  |
| M_S5_R |  |  | 0.432 |  |  |  |  |

| Agreement | MR1 | MR2 | MR4 | MR3 | MR5 | MR6 | MR7 |
| --- | --- | --- | --- | --- | --- | --- | --- |
| S_G1_A |  |  |  |  |  |  |  |
| S_G2_A |  |  |  |  | 0.491 |  |  |
| S_G3_A |  |  |  |  | 0.490 |  |  |
| S_G4_A |  |  |  |  | 0.619 |  |  |
| S_G5_A |  |  | 0.530 |  |  |  |  |
| S_S1_A |  |  |  |  |  |  |  |
| S_S2_A |  |  |  |  |  |  |  |
| S_S3_A |  |  |  |  |  |  |  |
| S_S4_A |  | 0.495 |  |  |  |  |  |
| S_S5_A | 0.596 |  | 0.483 |  |  |  |  |
| R_G1_A | 0.546 | 0.522 |  |  |  |  |  |
| R_G2_A | 0.878 |  |  |  |  |  |  |
| R_G3_A | 0.555 | 0.412 |  |  |  |  |  |
| R_G4_A | 0.800 |  |  |  |  |  |  |
| R_G5_A | 0.589 | 0.455 |  |  |  |  |  |
| R_S1_A | 0.864 |  |  |  |  |  |  |
| R_S2_A | 0.773 |  |  |  |  |  |  |
| R_S3_A | 0.642 |  |  |  |  |  |  |
| R_S4_A | 0.793 |  |  |  |  |  |  |
| R_S5_A | 0.516 |  |  |  |  |  |  |
| P_G1_A |  | 0.718 |  |  |  |  |  |
| P_G2_A |  | 0.738 |  |  |  |  |  |
| P_G3_A |  | 0.591 |  |  |  |  |  |
| P_G4_A |  | 0.731 |  |  |  |  |  |
| P_G5_A |  | 0.613 |  |  |  |  |  |
| P_S1_A |  | 0.724 |  |  |  |  |  |
| P_S2_A |  | 0.524 |  |  |  |  |  |
| P_S3_A |  | 0.785 |  |  |  |  |  |
| P_S4_A |  | 0.678 |  |  |  |  |  |
| P_S5_A |  | 0.781 |  |  |  |  |  |
| Pol_S1_A | 0.493 |  |  |  |  |  |  |
| Pol_S2_A |  |  |  |  |  |  |  |
| Pol_S3_A | 0.519 |  | 0.462 |  |  |  |  |
| Pol_S4_A |  |  |  |  |  |  |  |
| Pol_S5_A |  |  |  |  |  |  |  |
| Agreement | *MR1* | *MR2* | *MR4* | *MR3* | *MR5* | *MR6* | *MR7* |
| Pol_G1_A |  |  |  |  |  |  |  |
| Pol_G2_A |  |  | 0.459 |  |  |  |  |
| Pol_G3_A |  |  |  |  |  |  |  |
| Pol_G4_A |  |  |  |  |  | -0.618 |  |
| Pol_G5_A |  |  |  |  |  | 0.633 |  |
| M_G1_A |  |  |  |  |  |  |  |
| M_G2_A |  |  |  | 0.473 |  |  |  |
| M_G3_A |  |  |  | 0.582 |  |  |  |
| M_G4_A |  |  |  |  |  |  |  |
| M_G5_r_A |  |  |  |  |  |  |  |
| M_S1_A |  |  |  | 0.460 |  |  |  |
| M_S2_r_A |  |  |  |  |  |  | 0.409 |
| M_S3_A |  |  |  | 0.535 |  |  |  |
| M_S4_r_A |  |  |  |  |  |  |  |
| M_S5_A |  |  |  |  |  |  | 0.470 |
