# Supplementary material for: Beliefs are multidimensional and vary in stability over time - psychometric properties of the Beliefs and Values Inventory (BVI)
Source: PeerJ. 2019 Apr 25;7:e6819. doi: 10.7717/peerj.6819 (PMC6487186; doi:10.7717/peerj.6819)
Supplement: Appendix C — The Beliefs and Values Inventory (42 items) assess a more limited range of beliefs across themes of science, politics, the paranormal, religion, and morality, by dimensions of agreement, interest, and self-relevance. In addition, there are 5 control questions. [file peerj-07-6819-s003.docx]

**Beliefs and Values Inventory**

The Belief and Values Inventory aims to capture the agreement, perceived self-relevance, and interest that individuals place on the following statements. The questionnaire spans political, scientific, religious, paranormal, and moral statements, as well as including 5 control questions.

Participants are asked after each statement to rate their **Agreement, Self-Relevance, and Interest** on a scale of **1-10**.

| **Theme** |  | **Question** |
| --- | --- | --- |

| **Politics** |  | 1. The government or state has responsibility to look after the wellbeing of the population. |
| --- | --- | --- |
|  |  | 1. Democracy is the best way to govern a country. |
|  |  | 1. It’s good that a lot of countries and states have legalised abortion. |
|  |  | 1. It’s right to legally allow people of the same sex to get married. |
|  |  | 1. Women should be legally entitled to equal pay to men for the same work. |

| **Morality** |  | 1. It’s ok to hit or physically hurt someone for no reason if no one is around to know it happened. |
| --- | --- | --- |
|  |  | 1. Making a personal attack on someone on social media is ok if your identity is concealed. |
|  |  | 1. Saying something to cause emotional distress is fine if there aren’t any negative personal consequences. |
|  |  | 1. Running a red traffic light is fine if no one is around |
|  |  | 1. Drink-driving isn’t a problem as long as no one gets hurt. |
|  |  | 1. Not paying for a bus/train is fine as long as no one catches you. |

| **Science** |  | 1. Science will never fully understand the mysteries of the universe. |
| --- | --- | --- |
|  |  | 1. Reason and measurement are the only reliable basis for knowledge. |
|  |  | 1. Science will eventually give a more correct and reliable account of human behaviour than literary works such as novels, plays, and poetry. |
|  |  | 1. The scientific method is the best technique we have to understand the way we should live our lives. |
|  |  | 1. Science is our most powerful tool for improving human health. |
|  |  | 1. Evolutionary theory is the best explanation we have for the origin of species. |

| **Religion** |  | 1. Each human being has a soul or spirit which survives death. |
| --- | --- | --- |
|  |  | 1. God(s) can intervene in the world to influence natural processes |
|  |  | 1. Death is not the end of personal existence. |
|  |  | 1. God(s) only exists in the human imagination (r) |
|  |  | 1. Living a good life will mean rewards in the next. |
|  |  | 1. Prayer or religious practice is one of the most important ways of improving our lives. |
|  |  |  |
|  |  |  |
|  |  | 1. God can communicate with people, but people often mistake the message. 2. Practicing religion makes people more moral than atheists. 3. Religion should play a central role in society. |
|  |  | 1. If religions were properly practiced the world would be a better place. |

| **Paranormal** |  | 1. A person’s star sign gives important information about their character |
| --- | --- | --- |
|  |  |  |
|  |  | 1. The number 13 is unlucky. |
|  |  | 1. Tarot cards can genuinely predict the future. 2. Misalignment of chakras can cause ill health. |
|  |  | 1. Crystals can be used for healing. |
|  |  |  |
|  |  | 1. It is possible to communicate with people after they’ve died. |
|  |  |  |
|  |  |  |
|  |  | 1. Certain objects and actions can ward off bad luck. |
|  |  | 1. Some people have special powers to affect things at a distance. |
|  |  | 1. You can learn about your destiny through hidden signs in the world around us. |
|  |  | 1. Some people can affect the world around them purely through mental activity. |

| **Control Questions** |  | 1. The earth revolves around the sun. |
| --- | --- | --- |
|  |  | 1. Barack Obama was previously president of the USA. |
|  |  | 1. Murdering someone is punishable under criminal law in the UK. |
|  |  | 1. History is a study of the past. |
|  |  | 1. Australia is located in the southern hemisphere. |
