# Supplementary material for: Beliefs are multidimensional and vary in stability over time - psychometric properties of the Beliefs and Values Inventory (BVI)
Source: PeerJ. 2019 Apr 25;7:e6819. doi: 10.7717/peerj.6819 (PMC6487186; doi:10.7717/peerj.6819)
Supplement: Appendix D [file peerj-07-6819-s004.docx]

**A**

| ID | type | ICC | F | df1 | df2 | p | lower bound | upper bound | TimePoint |
| --- | --- | --- | --- | --- | --- | --- | --- | --- | --- |
| S_A | ICC3 | 0.8217584 | 10.220726 | 97 | 97 | 0.00E+00 | 0.7451811 | 0.8769456 | 48 Hours |
| S_I | ICC3 | 0.8663655 | 13.966195 | 97 | 97 | 0.00E+00 | 0.8069215 | 0.9084356 | 48 Hours |
| S_R | ICC3 | 0.7976325 | 8.88301 | 97 | 97 | 0.00E+00 | 0.7123268 | 0.8597143 | 48 Hours |
| PA_A | ICC3 | 0.8999093 | 18.981873 | 97 | 97 | 0.00E+00 | 0.8542209 | 0.9318052 | 48 Hours |
| PA_I | ICC3 | 0.8776624 | 15.348199 | 97 | 97 | 0.00E+00 | 0.8227663 | 0.9163355 | 48 Hours |
| PA_R | ICC3 | 0.8426338 | 11.709207 | 97 | 97 | 0.00E+00 | 0.773912 | 0.8917416 | 48 Hours |
| R_A | ICC3 | 0.9620612 | 51.716544 | 97 | 97 | 0.00E+00 | 0.9439057 | 0.9744179 | 48 Hours |
| R_I | ICC3 | 0.8631012 | 13.609333 | 97 | 97 | 0.00E+00 | 0.8023589 | 0.9061473 | 48 Hours |
| R_R | ICC3 | 0.8436825 | 11.794474 | 97 | 97 | 0.00E+00 | 0.7753629 | 0.8924822 | 48 Hours |
| Po_A | ICC3 | 0.9112714 | 21.54064 | 97 | 97 | 0.00E+00 | 0.8704157 | 0.9396615 | 48 Hours |
| Po_I | ICC3 | 0.8544446 | 12.740477 | 97 | 97 | 0.00E+00 | 0.7902937 | 0.9000667 | 48 Hours |
| Po_R | ICC3 | 0.8594204 | 13.22682 | 97 | 97 | 0.00E+00 | 0.7972227 | 0.903564 | 48 Hours |
| M_A | ICC3 | 0.6069834 | 4.088844 | 97 | 97 | 1.37E-11 | 0.46522 | 0.7183898 | 48 Hours |
| M_I | ICC3 | 0.7663164 | 7.55858 | 97 | 97 | 0.00E+00 | 0.6702314 | 0.8371349 | 48 Hours |
| M_R | ICC3 | 0.6767327 | 5.186831 | 97 | 97 | 7.44E-15 | 0.5531321 | 0.7711829 | 48 Hours |

**B**

| ID | type | ICC | F | df1 | df2 | p | lower | upper | TimePoint |
| --- | --- | --- | --- | --- | --- | --- | --- | --- | --- |
| S_A | ICC3 | 0.7701318 | 7.700636 | 68 | 68 | 3.33E-15 | 0.6532613 | 0.8511567 | 3.5 Months |
| S_I | ICC3 | 0.7950353 | 8.757778 | 68 | 68 | 1.11E-16 | 0.688599 | 0.8679371 | 3.5 Months |
| S_R | ICC3 | 0.7135546 | 5.982135 | 68 | 68 | 2.07E-12 | 0.5748282 | 0.8124088 | 3.5 Months |
| PA_A | ICC3 | 0.8350481 | 11.124743 | 68 | 68 | 0.00E+00 | 0.7464551 | 0.8945541 | 3.5 Months |
| PA_I | ICC3 | 0.8391389 | 11.433087 | 68 | 68 | 0.00E+00 | 0.7524467 | 0.8972518 | 3.5 Months |
| PA_R | ICC3 | 0.8210743 | 10.177824 | 68 | 68 | 0.00E+00 | 0.7260965 | 0.8853063 | 3.5 Months |
| R_A | ICC3 | 0.8942085 | 17.905118 | 68 | 68 | 0.00E+00 | 0.8345245 | 0.9331501 | 3.5 Months |
| R_I | ICC3 | 0.8229238 | 10.294569 | 68 | 68 | 0.00E+00 | 0.7287815 | 0.8865332 | 3.5 Months |
| R_R | ICC3 | 0.805244 | 9.26926 | 68 | 68 | 0.00E+00 | 0.7032325 | 0.8747681 | 3.5 Months |
| Po_A | ICC3 | 0.8316857 | 10.882529 | 68 | 68 | 0.00E+00 | 0.7415412 | 0.8923335 | 3.5 Months |
| Po_I | ICC3 | 0.7509371 | 7.0301 | 68 | 68 | 3.53E-14 | 0.6263676 | 0.8381091 | 3.5 Months |
| Po_R | ICC3 | 0.693159 | 5.518034 | 68 | 68 | 1.47E-11 | 0.5471653 | 0.798223 | 3.5 Months |
| M_A | ICC3 | 0.6108659 | 4.139617 | 68 | 68 | 9.79E-09 | 0.4386977 | 0.7397768 | 3.5 Months |
| M_I | ICC3 | 0.6934352 | 5.523906 | 68 | 68 | 1.43E-11 | 0.5475377 | 0.7984158 | 3.5 Months |
| M_R | ICC3 | 0.6497883 | 4.710831 | 68 | 68 | 5.78E-10 | 0.4893821 | 0.7676648 | 3.5 Months |
