# Supplementary material for: Beliefs are multidimensional and vary in stability over time - psychometric properties of the Beliefs and Values Inventory (BVI)
Source: PeerJ. 2019 Apr 25;7:e6819. doi: 10.7717/peerj.6819 (PMC6487186; doi:10.7717/peerj.6819)
Supplement: Appendix E [file peerj-07-6819-s005.docx]

**A**

| ID | type | ICC | F | df1 | df2 | p | lower bound | upper bound | TimePoint |
| --- | --- | --- | --- | --- | --- | --- | --- | --- | --- |
| S_A | ICC3 | 0.7530119 | 7.097557 | 97 | 97 | 0.00E+00 | 0.6525326 | 0.8274686 | 48 Hours |
| S_I | ICC3 | 0.8514659 | 12.464921 | 97 | 97 | 0.00E+00 | 0.7861535 | 0.8979702 | 48 Hours |
| S_R | ICC3 | 0.8085382 | 9.445946 | 97 | 97 | 0.00E+00 | 0.7271318 | 0.867521 | 48 Hours |
| PA_A | ICC3 | 0.8981928 | 18.644966 | 97 | 97 | 0.00E+00 | 0.851782 | 0.9306157 | 48 Hours |
| PA_I | ICC3 | 0.8783008 | 15.433957 | 97 | 97 | 0.00E+00 | 0.8236642 | 0.916781 | 48 Hours |
| PA_R | ICC3 | 0.8445059 | 11.862228 | 97 | 97 | 0.00E+00 | 0.7765026 | 0.8930635 | 48 Hours |
| R_A | ICC3 | 0.9637248 | 54.134115 | 97 | 97 | 0.00E+00 | 0.9463436 | 0.9755464 | 48 Hours |
| R_I | ICC3 | 0.866135 | 13.94042 | 97 | 97 | 0.00E+00 | 0.806599 | 0.9082741 | 48 Hours |
| R_R | ICC3 | 0.859999 | 13.285608 | 97 | 97 | 0.00E+00 | 0.7980294 | 0.9039702 | 48 Hours |
| Po_A | ICC3 | 0.8537255 | 12.672923 | 97 | 97 | 0.00E+00 | 0.7892936 | 0.8995607 | 48 Hours |
| Po_I | ICC3 | 0.8681777 | 14.171944 | 97 | 97 | 0.00E+00 | 0.8094575 | 0.9097049 | 48 Hours |
| Po_R | ICC3 | 0.8605654 | 13.343641 | 97 | 97 | 0.00E+00 | 0.7988194 | 0.9043679 | 48 Hours |
| M_A | ICC3 | 0.5170883 | 3.141543 | 97 | 97 | 2.13E-08 | 0.3558958 | 0.6484015 | 48 Hours |
| M_I | ICC3 | 0.7099302 | 5.894893 | 97 | 97 | 1.11E-16 | 0.5959641 | 0.7958619 | 48 Hours |
| M_R | ICC3 | 0.6765445 | 5.183231 | 97 | 97 | 7.55E-15 | 0.5528911 | 0.7710422 | 48 Hours |

**B**

| ID | type | ICC | F | df1 | df2 | p | lower bound | upper bound | TimePoint |
| --- | --- | --- | --- | --- | --- | --- | --- | --- | --- |
| S_A | ICC3 | 0.6908382 | 5.469105 | 68 | 68 | 1.82E-11 | 0.5440376 | 0.7966014 | 3.5 Months |
| S_I | ICC3 | 0.7855672 | 8.326933 | 68 | 68 | 4.44E-16 | 0.6751041 | 0.8615769 | 3.5 Months |
| S_R | ICC3 | 0.7018549 | 5.708144 | 68 | 68 | 6.49E-12 | 0.5589208 | 0.8042855 | 3.5 Months |
| PA_A | ICC3 | 0.8358576 | 11.184545 | 68 | 68 | 0.00E+00 | 0.7476397 | 0.8950883 | 3.5 Months |
| PA_I | ICC3 | 0.8373115 | 11.293434 | 68 | 68 | 0.00E+00 | 0.7497685 | 0.8960473 | 3.5 Months |
| PA_R | ICC3 | 0.8234935 | 10.331031 | 68 | 68 | 0.00E+00 | 0.7296093 | 0.886911 | 3.5 Months |
| R_A | ICC3 | 0.8954656 | 18.132455 | 68 | 68 | 0.00E+00 | 0.8364295 | 0.9339605 | 3.5 Months |
| R_I | ICC3 | 0.8140472 | 9.755417 | 68 | 68 | 0.00E+00 | 0.7159212 | 0.8806365 | 3.5 Months |
| R_R | ICC3 | 0.8241413 | 10.372769 | 68 | 68 | 0.00E+00 | 0.7305507 | 0.8873404 | 3.5 Months |
| Po_A | ICC3 | 0.7603744 | 7.346355 | 68 | 68 | 1.13E-14 | 0.6395533 | 0.8445367 | 3.5 Months |
| Po_I | ICC3 | 0.7591093 | 7.30252 | 68 | 68 | 1.32E-14 | 0.6377815 | 0.8436764 | 3.5 Months |
| Po_R | ICC3 | 0.7241746 | 6.250964 | 68 | 68 | 6.97E-13 | 0.5893589 | 0.8197492 | 3.5 Months |
| M_A | ICC3 | 0.5282494 | 3.239529 | 68 | 68 | 1.30E-06 | 0.3346267 | 0.6790767 | 3.5 Months |
| M_I | ICC3 | 0.5416134 | 3.36313 | 68 | 68 | 6.44E-07 | 0.3511469 | 0.6890373 | 3.5 Months |
| M_R | ICC3 | 0.5640153 | 3.587317 | 68 | 68 | 1.84E-07 | 0.3791081 | 0.7056103 | 3.5 Months |
